# Pontine control of rapid eye movement sleep and fear memory

**DOI:** 10.1111/cns.14123

**Published:** 2023-02-16

**Authors:** Yu Jun Wen, Wen Jia Yang, Chun Ni Guo, Mei Hong Qiu, Daniel Kroeger, Jian Guo Niu, Shu Qin Zhan, Xi Fei Yang, Barbara Gisabella, Ramalingam Vetrivelan, Jun Lu

**Affiliations:** ^1^ Ningxia Key Laboratory of Craniocerebral Diseases, Department of Anatomy, Histology and Embryology, School of Basic Medicine Ningxia Medical University Yinchuan Ningxia China; ^2^ Department of Neurology Beth Israel Deaconess Medical Center and Harvard Medical School Boston Massachusetts USA; ^3^ Shanghai Yueyang Integrated Medicine Hospital Shanghai China; ^4^ Department of Neurology, Shanghai First People's Hospital Shanghai Jiaotong University Shanghai China; ^5^ Department of Neurobiology, School of Basic Medical Science, Shanghai Medical College Fudan University Shanghai China; ^6^ Department of Anatomy, Physiology & Pharmacology, College of Veterinary Medicine Auburn University Auburn Alabama USA; ^7^ Department of Neurology, Xuanwu Hospital Capital Medical University Beijing China; ^8^ Shenzhen Center for Disease Control and Prevention Shenzhen Guangdong China; ^9^ Department of Psychiatry and Human Behavior University of Mississippi Medical Center Jackson Mississippi USA; ^10^ Stroke Center, Department of Neurology 1^st^ Hospital of Jilin University Changchun Jilin China

**Keywords:** fear memory, glutamate, hippocampus, medial septum, neural circuit, REM sleep, SLD

## Abstract

**Aims:**

We often experience dreams of strong irrational and negative emotional contents with postural muscle paralysis during rapid eye movement (REM) sleep, but how REM sleep is generated and its function remain unclear. In this study, we investigate whether the dorsal pontine sub‐laterodorsal tegmental nucleus (SLD) is necessary and sufficient for REM sleep and whether REM sleep elimination alters fear memory.

**Methods:**

To investigate whether activation of SLD neurons is sufficient for REM sleep induction, we expressed channelrhodopsin‐2 (ChR2) in SLD neurons by bilaterally injecting AAV1‐hSyn‐ChR2‐YFP in rats. We next selectively ablated either glutamatergic or GABAergic neurons from the SLD in mice in order to identify the neuronal subset crucial for REM sleep. We finally  investigated the role of REM sleep in consolidation of fear memory using rat model with complete SLD lesions.

**Results:**

We demonstrate the sufficiency of the SLD for REM sleep by showing that photo‐activation of ChR2 transfected SLD neurons selectively promotes transitions from non‐REM (NREM) sleep to REM sleep in rats. Diphtheria toxin‐A (DTA) induced lesions of the SLD in rats or specific deletion of SLD glutamatergic neurons but not GABAergic neurons in mice completely abolish REM sleep, demonstrating the necessity of SLD glutamatergic neurons for REM sleep. We then show that REM sleep elimination by SLD lesions in rats significantly enhances contextual and cued fear memory consolidation by 2.5 and 1.0 folds, respectively, for at least 9 months. Conversely, fear conditioning and fear memory trigger doubled amounts of REM sleep in the following night, and chemo‐activation of SLD neurons projecting to the medial septum (MS) selectively enhances hippocampal theta activity in REM sleep; this stimulation immediately after fear acquisition reduces contextual and cued fear memory consolidation by 60% and 30%, respectively.

**Conclusion:**

SLD glutamatergic neurons generate REM sleep and REM sleep and SLD via the hippocampus particularly down‐regulate contextual fear memory.

## Graphical abstract

Pontine sub‐laterodorsal tegmental nucleus (SLD) is necessary and sufficient for rapid‐eye‐movement (REM) sleep, and REM sleep down‐regulates fear memory.

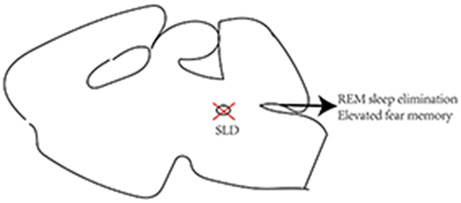

## 1 INTRODUCTION

Early complete brainstem transection studies demonstrated that the pons is necessary for rapid eye movement (REM) sleep by Jouvet and colleagues.^1^ They then first identified the area ventral to the locus coeruleus to be the most‐sensitive site for REM sleep induction by carbachol injection in cats.^2^, ^3^ A homologous structure locates slightly rostral and ventral to the caudal laterodorsal tegmental nucleus in rats and thus is named as the sub‐laterodorsal tegmental nucleus (SLD).^4^, ^5^ Electrolytic or chemical lesions of this pontine region in cats or rats produced as much as 50% reduction in REM sleep amounts and induced REM sleep behavior disorder (RBD)‐like movements.^5^, ^6^, ^7^, ^8^ The SLD contains glutamatergic and GABAergic neurons. Selective deletion of SLD glutamate neurotransmission, but not GABA neurotransmission in mice, recapitulates REM sleep reduction and RBD‐like movements by SLD lesions in rats, indicating a crucial role for SLD glutamatergic neurons in REM sleep.^9^ However, neither SLD lesions nor deletion of glutamate transmission completely eliminated REM sleep,^5^, ^9^ which in conjunction of existence of RBD‐like movements after lesions or glutamate deletion suggests incomplete lesions, and it raises an important question of whether the SLD is necessary and sufficient for REM sleep. The putative functions of REM sleep include learning and memory process, particularly negative emotion memories.^10^, ^11^ However, it is controversial whether REM sleep promotes consolidation or de‐potentiation of such memories because REM sleep deprivation studies in animals^12^, ^13^, ^14^, ^15^, ^16^ and humans^13^, ^17^, ^18^, ^19^ appear to support both claims. These discrepancies could be potentially due to inherent stress of various levels associated with REM sleep deprivation procedures, which depending on stress levels may exert contrast effects on hippocampal memory functions.^20^, ^21^ Therefore, it is crucial to evaluate the negative emotional memory processing in REM sleep loss by stress‐free animal model.

To address the questions raised, we optogenetically activated the SLD neurons to test the sufficiency for REM sleep induction, ablated SLD neurons or glutamatergic neurons using a novel diphtheria toxin‐based lesion approach, and investigated whether REM sleep is eliminated and finally tested whether REM sleep elimination affects fear memory.

## 2 MATERIALS AND METHODS

### 2.1 Animals

Adult male Sprague–Dawley rats (Harlan, USA; 280–320 g) were used for all rat experiments. For mouse experiments, we used adult male Vglut2‐Cre mice, Vgat‐Cre mice, and their wild‐type littermates (24–28 g). All animals were maintained on standard vivarium conditions (L:D = 12:12; lights‐on at 07:00 and 22.0 ± 1.0°C) with free access to food and water. All protocols were approved by the Institutional Animal Care and Use Committee of Beth Israel Deaconess Medical Center.

### 2.2 Optogenetic experiments

Under anesthesia (ketamine 80 mg/kg + xylazine 10 mg/kg mixture), rats were stereotaxically injected with 20 μL of AAV1‐hSyn‐ChR2[H134R]‐YFP bilaterally into the SLD (AP: −9.25 mm, ML: ±0.8 mm, DV: −6.3 mm, Paxinos & Watson) and implanted with optical fibers (200 μm diameter, 0.39 N/A, Thorlabs) targeting 200 μm above the SLD and electrodes for recording electroencephalogram (EEG) and electromyogram (EMG). After 2 weeks of post‐surgical recovery, we acclimated the rats for 1 week to the EEG/EMG recording cables and the optical fiber leads. We then performed EEG/EMG and video recordings at ZT 07 for 5 h. One hour after the onset of recordings, we applied laser light stimulation (10 ms pulses at 10 Hz for 25 s every 10 min for 4 h) using a DPSS laser (473 nm wavelength; Shanghai Laser & Optics Century Co., Ltd). Importantly, to avoid stimulating nearby neurons in, for example, the parabrachial nucleus or locus coeruleus, we reduced the light power output of our light source to produce only ~0.15 mW light at the fiber tip (~1.2 mW/mm^2^ irradiance, i.e., near the minimum of light required for ChR2 activation), limiting the photo‐activation to neurons within a very close range of the optical fiber tip. After all data collection, two rats underwent photostimulation (10 Hz; 10 s on 20 s off) for 2 h and then were deeply anesthetized (chloral hydrate, 500 mg/kg) and transcardially perfused with 10% formalin. Brains were sectioned at 40 μm in four series and one series was immunolabeled for YFP (to label the ChR2‐expressing neurons) and cFos (to label the neurons activated by photostimulations) as descried previously.^22^ Histological analysis showed that three of the seven rats had bilateral off‐target viral injections and thus served as “light‐only” control animals.

### 2.3 SLD lesions and sleep–wake recording in rats and mice

For SLD ablations in rats, we anesthetized rats by ketamine and xylazine and injected a mixture of AAV8‐Cre‐GFP (University of North Carolina Vector Core) and a Cre‐dependent AAV vector encoding for diphtheria toxin A subunit (rAAV8‐EF1α‐mCherry‐Flex‐DTA; “AAV‐DTA”; University of North Carolina Vector Core) bilaterally into the SLD (AP: −9.25 mm, DV: −6.3 mm, ML: ±0.8 mm). Injections of this mixture lead to the expression of Cre and consequently DTA, which kills all the neurons containing Cre and DTA.^23^ Control rats received bilateral injections of AAV8‐GFP into SLD.

For selective ablation of glutamatergic or GABAergic neurons in the SLD, we used Cre‐dependent DTA.^24^ We streotaxically injected Cre‐dependent AAV‐DTA into the SLD (AP: −5.3 mm, ML: ±0.7 mm, DV: −3.2 mm) of the Vglut2‐IRES‐Cre::L10‐GFP mice or Vgat‐IRES‐Cre::L10‐GFP mice, respectively. This AAV‐DTA would transfect all the neurons that express mCherry in the injected sites but kills the neurons containing Cre‐recombinase (i.e., VGLUT2 or VGAT neurons).

After AAV‐DTA injections, rats and mice were implanted with four EEG screw electrodes (two frontal and two parietal electrodes) and two flexible EMG wire electrodes (Plastics One), and the entire assembly was secured onto skull with dental cement. Three weeks after surgeries, animals were housed individually in transparent barrels in an insulated soundproof recording chamber (maintained at similar conditions as the vivarium; 22 ± 1°C and on a 12‐h light/dark cycle and unlimited access to food and water) and habituated to the recording cable for at least 1 day. Then, EEG/EMG and time‐locked video were recorded for 48 h. Sleep–wake stages were manually scored to wake, non‐REM (NREM), or REM sleep in 10‐s bins in mice and 12 s in rats (SleepSign, Kissei Comtek). For the quantification of RBD‐like movements, we calculated the number and duration of phasic movements in NREM sleep and REM sleep and transitions into wake based on video and EEG/EMG signals. We only counted twitches with obvious behaviors and EMG changes. In some cases, animals may have EMG changes but with only very small twitches; those were not included in the analysis. Control rats displayed 0–3 twitches or jerking movements per day, while rats with partial SLD lesions displayed a greater number and more complex movements (such as running) in addition to simple twitches. As these complex behaviors lasted longer than brief muscle twitches, long duration RBD‐like movements in this study may represent more complex behaviors. After completion of all data collection, rats and mice were deeply anesthetized (chloral hydrate, 500 mg/kg) and transcardially perfused with saline and 10% formalin (Sigma). Brains were sectioned at 40 μm in four series (rats) or 30 μm in four series (mice). Two series from rat brains were immunolabeled for NeuN or Nissl‐stained to verify the lesions. For the verification of lesion sites in mice, one series of sections were mounted onto slides and the GFP^+^ neurons (representing glutamatergic or GABAergic neurons in the respective mice) and mCherry^+^ neurons (representing the non‐glutamatergic or GABAergic neurons not killed by the DTA in the respective mice) were visualized under the fluorescent microscope (VS120 slide scanner microscope).

### 2.4 Fear memory test

To examine the long‐term effects of SLD lesions on fear memory, we used a fear conditioning chamber and the analysis program from Med Associates system (NIR‐018MD) for this test. On day 1, rats were placed in the chamber with interior white light and 1.0% acetic acid (context A) for 180 s. Rats then received three tones and foot‐shocks (tone, 2000 Hz, 85 dB, 18 s; shock, 0.54 V, 2 s) with 30‐s interval. On day 2, rats were placed in the same box with a different interior environment and odor (red light and pine oil odor, context B) for 9 mins and 50 s. On day 3, rats were placed in the box with same context A as on day 1 with a 120‐s pre‐tone period before receiving 5 × 20 s tone with 30 s intervals, but no footshocks. On day 7 (or week 1), rats were placed again in the context A and (after 120 s acclimation) they were presented with 180 s of tone. We repeated day 7 procedure in week 2–6 and then week 25. These procedures were conducted between 10:00 and 12:00. The percentage of freezing time was determined by the threshold of freezing (i.e., the value of the motion index below which no movement is detectable), and the percentage of observations below this threshold was calculated for the times of interest (interval before the tone, tone presentation, and interval after the tone) on days 1–3 and weeks 1–25. Freezing to pre‐tone period is considered as contextual fear response, while freezing to tone is considered as cued fear response.

### 2.5 Chemo‐activation of SLD projections to the medial septum (MS)

Under anesthesia as described above, we placed bilateral injections of a retrograde viral vector AAV6‐Cre (100 nL), which is taken up by the axonal terminals and retrogradely transported to the cell bodies into the MS (AP: +0.5 mm, ML: 0 mm, DV: −6.6 mm) and a Cre‐dependent AAV8‐hM3Dq‐mCherry (100 nL) into the SLD in six rats. EEG/EMG electrodes were then implanted in those rats.

After 3 weeks of post‐surgical recovery and habituation (as above), we injected 0.1 mg/kg clozapine‐N‐oxide (CNO, hM3 agonist to activate the hM3‐expressing neurons) or the vehicle (saline, i.p.) at 10:00 am and recorded sleep–wake for 48 h. Then, to test the effects of SLD‐MS pathway activation on fear memory, CNO was injected immediately after rats received fear training on day 1 of fear conditioning paradigm (see Section 2.4). Day 2 testing in altered contexts (context B) and day 3 testing with only tones were then performed in the same context (context A) of day 1. The same protocol was used in control rats receiving sham surgery. Upon completion of data collection, all rats were injected with CNO (0.1 mg/kg) and perfused after 2 h. One series of sections were immuno‐stained for mCherry (to label the hM3 expressing neurons) and cFos (to label the neurons activated by CNO) and a second series was immunolabeled for Cre (for labeling the neurons transduced by AAV6‐Cre at the injection site).

### 2.6 Statistical analysis

Data were presented as a mean ± standard error of the mean. For comparisons between SLD lesions vs. sham control in sleep–wake amounts and fear memory as well as CNO vs. saline in fear memory, analysis of variance (ANOVA) and unpaired *t*‐test were used. For FFT analysis and sleep amounts of saline and CNO injection as well as fear‐induced sleep changes in the same rats, paired *t*‐test was applied. Since FFT baseline values were very different, we compared ratio of CNO/saline vs. saline/saline by using two‐way ANOVA. The Kolmogorov–Smirnov test revealed that the distribution of all data was normal. *p* < 0.05 was considered statistically significant.

## 3 RESULTS

### 3.1 Photoactivation of SLD neurons promotes transitions from NREM sleep to REM sleep

**FIGURE 1:**
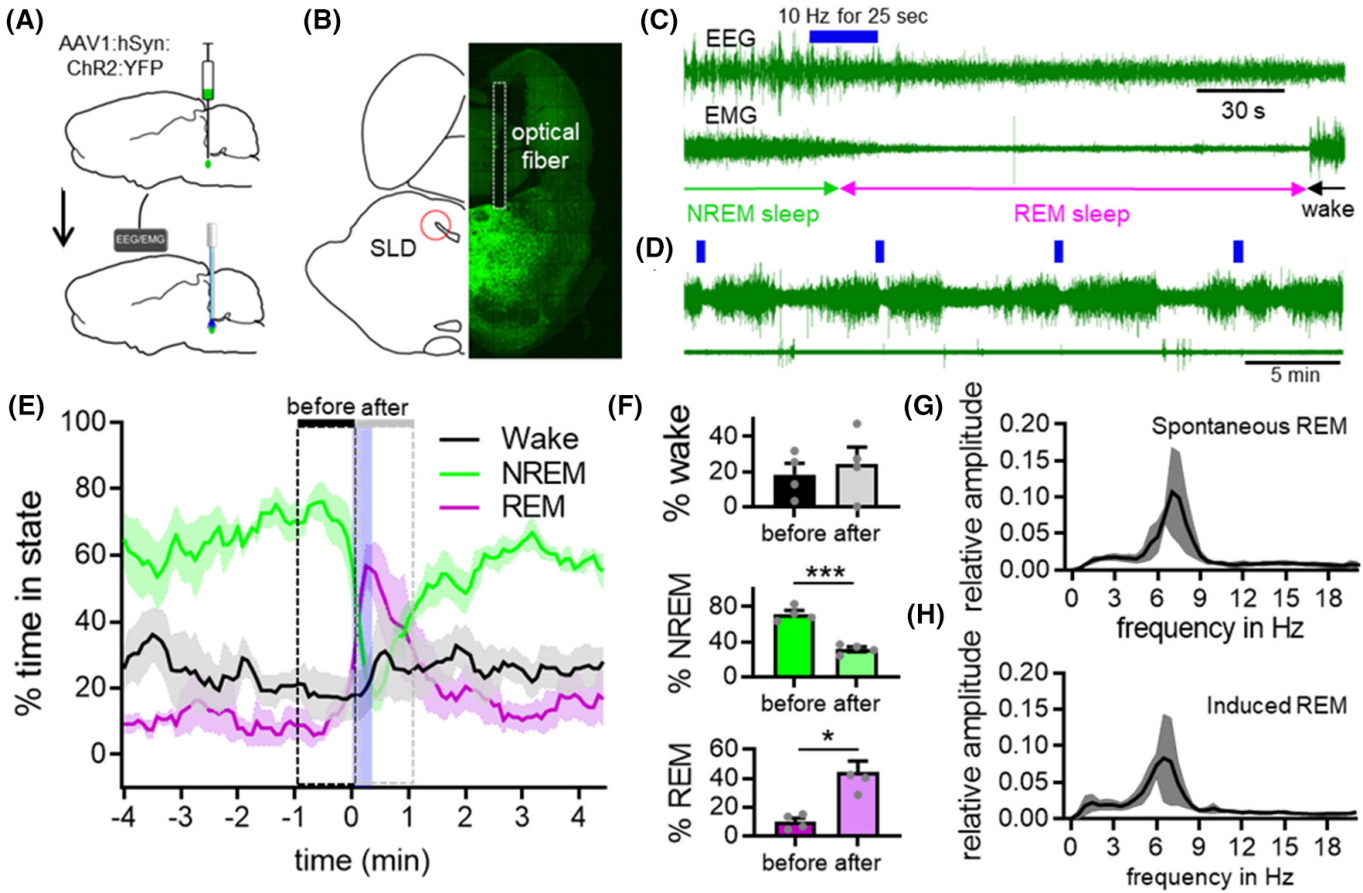
**Optogenetic stimulation of SLD neurons promotes transitions from NREM sleep to REM sleep**. (A) Bilateral injection of AAV1‐hSyn‐ChR2‐YFP into the SLD and implantation of optical fibers in four rats. (B) YFP expression in virally transduced neurons and fiber placement in the SLD. (C) Representative trace of photoactivation of SLD neurons during NREM sleep (10 Hz; 10 ms pulses for 25 s) inducing transitions from NREM sleep to REM sleep. (D) Sample traces of automated stimulations every 10 min. (E) Sleep–wake state changes before, during, and after automated stimulation applied every 10 min for 4 h. (F) Photostimulation increased REM sleep during the one minute‐period after stimulation onset, as compared to 1 min before the stimulation (*p* = 0.0132, 2‐tailed *t*‐test), and decreased NREM sleep (*p* = 0.0009, 2‐tailed *t*‐test). FFT during spontaneous REM sleep (G) and REM sleep after photostimulation (H). Data were represented as mean ± SEM and analyzed by paired *t*‐test, *N* = 4, *p < 0.05, ***p < 0.001.

To investigate whether activation of SLD neurons is sufficient for REM sleep induction, we expressed channelrhodopsin‐2 (ChR2) in SLD neurons by bilaterally injecting 20 nL of a non‐Cre‐dependent adeno‐associated viral vectors (AAV) coding for ChR2 and yellow fluorescent protein (YFP) (AAV1‐hSyn‐ChR2[H134R]‐YFP) in rats. We also implanted optical fibers (~0.2 mm above the injection sites) as well as EEG/EMG electrodes (*N* = 4, Figure 1A,B). In addition, three rats with off‐target viral injections served as “light only” controls to ascertain that SLD stimulation with light in the absence of ChR2 has no effects on sleep–wake behavior. To assess whether light application in the SLD activates ChR2‐expressing neurons, we applied 2 h of photostimulation or sham stimulation (no light) and perfused the rats (*N* = 2 in each group). Photostimulation, but not sham stimulation, induced cFos expression in ChR2‐transduced neurons (YFP^+^) in the SLD immediately ventral to the optical fiber tip, confirming their activation (Figure S1A–C). We also mapped the viral injection sites and optical fiber placement in all rats (Figure S1D). Notice that despite small injection volume, viral transfection was spread larger than the SLD. However, because the probes were just above the SLD and light could not penetrate far, the stimulation was within the SLD confirmed by cFos expression.

To assess the effects of SLD activation on vigilance states, we acutely photoactivated SLD neurons with 10 Hz blue laser light (473 nm; 10 ms pulses) for 25 s every 10 min, irrespective of vigilance states, for multiple 4 h recording sessions starting at ZT 8 (2:00 pm; Figure 1C,D), and compared the percent time spent in each state during 1 min before and after the laser onset. REM sleep was increased (44.1 ± 7.7% post‐activation vs. 9.9 ± 2.6% prior to activation) and NREM sleep was decreased (31.6 ± 2.8% post‐activation vs. 71.7 ± 4.2% prior to activation) during the post‐stimulation period (Figure 1E,F). REM sleep levels decreased slowly and reached baseline levels after about 100 s. Photo‐activations specifically during wake or REM sleep did not result in state changes (Figure S1E,F), indicating that SLD activation only promotes transition from NREM sleep to REM sleep. EEG spectra of REM sleep states by SLD stimulation were similar (Figure 1G,H) to spontaneous REM sleep and the animals woke up briefly after the stimulation‐induced REM sleep, but transitioned into NREM sleep quickly, indicating natural state transitions. We also did not observe any sleep–wake changes in the “light only” control rats.

### 3.2 SLD lesions (SLDx) eliminate REM sleep in rats

**FIGURE 2:**
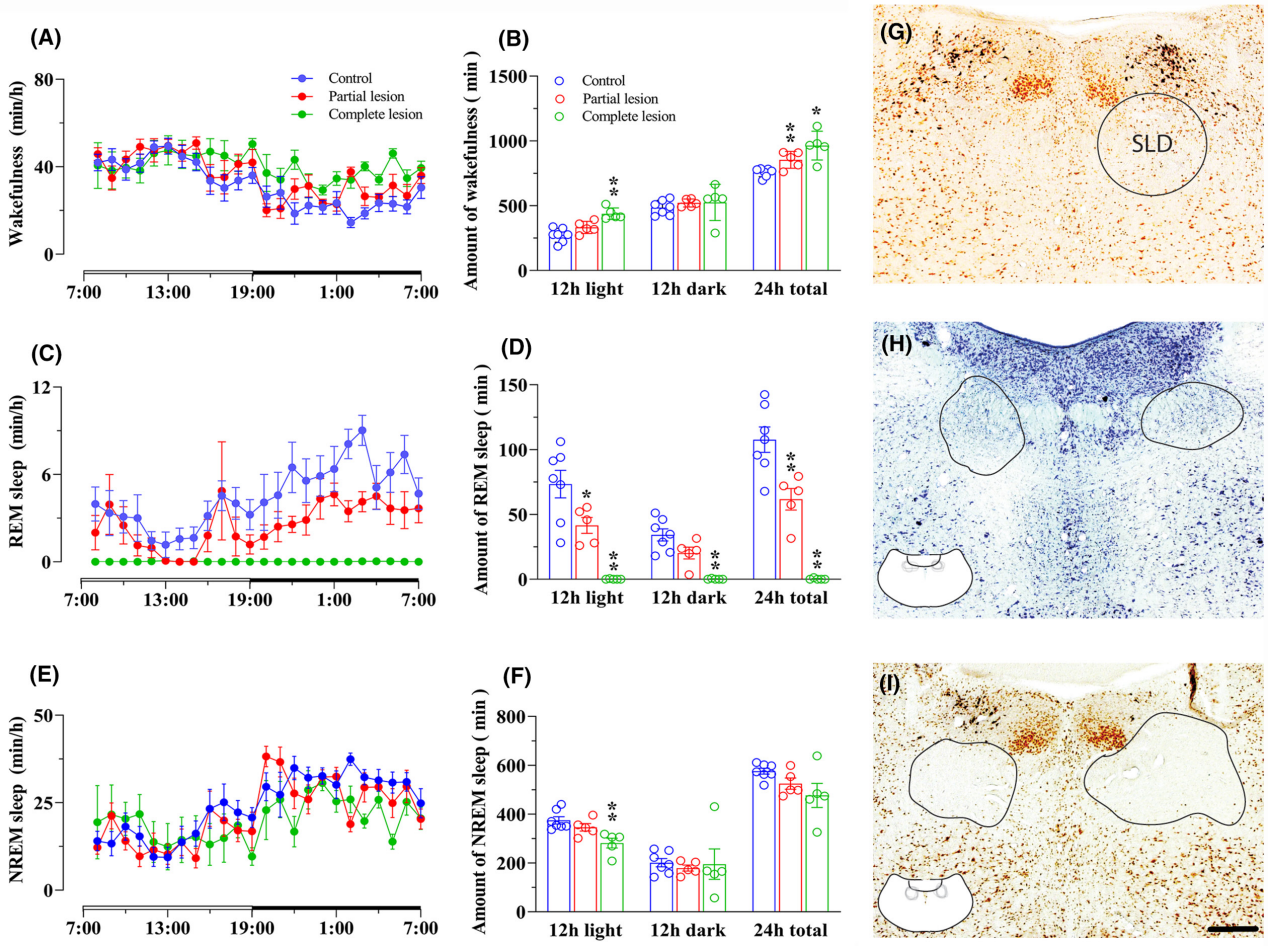
**SLD lesion eliminates REM sleep in rat.** To investigate whether the SLD is necessary for REM sleep, we ablated SLD neurons by bilateral injections of AAV‐DTA and examined sleep–wake parameters in lesioned vs. control rats. We obtained partial lesion group (*N* = 5), complete lesion group (*N* = 5), and control group (*N* = 7). Hourly wake, REM sleep, and NREM sleep amounts across 24 h are shown in A, C, and E while total amounts of wake, REM sleep, and NREM sleep in 24, 12 dark and 12 h light period are shown in B, D, and F. Histology of SLD location, partial lesion, and complete lesion are shown in G, H, and I. Complete SLD lesions eliminate REM sleep, significantly reduce NREM sleep during the light period, and significantly increase wake amounts in 24 h and light period. Partial SLD lesions have less effect on sleep–wake than complete SLD lesions, but produce RBD‐like movements (Figure S3). Hourly wake, REM, and NREM sleep amounts were analyzed using two‐way ANOVA, and total amounts of each stage were analyzed using one‐way ANOVA, followed by Bonferroni's post hoc test, *p < 0.05, **p < 0.01. Scale bar = 200 μm.

To assess whether the SLD is necessary for REM sleep, we intentionally ablated the SLD with minimal intrusion to the surrounding regions using a diphtheria toxin‐based approach with relatively small doses. Rats (*N* = 10) were stereotaxically injected with 90 nL of a 1:1 mixture of two AAV—an AAV containing Cre recombinase (AAV‐Cre‐GFP) and a Cre‐dependent AAV containing diphtheria toxin subunit A (AAV‐DTA‐mCherry) bilaterally into the SLD. This particular dose was tested and verified to kill the SLD with minimal lesions of surrounding structures. Because of minimal lesion areas, any injections were slightly off of the targets, and lesions became partial. Rats injected with AAV8‐GFP (90 nL; *N* = 7) into the SLD served as sham controls. All rats were then implanted with EEG/EMG electrodes.^25^ Three weeks after surgery, we recorded EEG/EMG with time‐locked video for 24 h in each rat. Rats were then perfused with 10% formalin and the harvested brains sliced into four series of 40 μm sections. The lesions were verified by Nissl‐staining or NeuN immuolabeling (a biomarker for neurons). Because lesions were small, we ended up with five rats of complete lesions and five rats of partial lesions. The complete lesions were mostly confined to the SLD region avoiding the caudal laterodorsal tegmental nucleus (LDT) and the medial parabrachial nucleus (PB) (Figure 2, G‐I). We grouped animals as control (Figure 2G), partial lesions (at least 50% neuronal loss on either side, Figure 2H), and complete lesions (lesions covering the entire SLD bilaterally, Figure 2I).

SLDx rats (*N* = 5) were in good health but showed restless movements in an open space and aggression, low body weight gain (growth rate: control, 8%; lesion, 2%). They displayed almost no REM sleep during the entire 24‐h recording period and all the NREM sleep bouts transitioned into wake (Figure 2D, Figure S2). Very short residual REM sleep of a few seconds was occasionally observed in some SLDx rats, and the REM sleep was always immediately transited to wakefulness. Given the unique EEG feature and extreme high ratio of theta power/delta power in REM sleep that was much higher than wake or NREM sleep, REM sleep was distinctly identified. In addition, these rats exhibited fragmented sleep pattern characterized by more frequent NREM‐wake transitions and shorter NREM bouts (Figure S2). The daily wake amounts were significantly increased in these rats (66.8 ± 3.4% vs. 52.5 ± 0.8% in controls; *p* < 0.05) with a non‐significant decrease in NREM sleep amounts. As REM sleep was completely absent, REM sleep without atonia or RBD‐like movements was not observed (Figure 2A–F). Finally, SLDx reduced circadian rhythm of sleep–wake state (Figure 2A–F).

Rats with partial SLDx (*N* = 5) exhibited a significant reduction in REM sleep as well as an increase in wake and reduction in circadian rhythm of wake (Figure 2A–F). Similar to our previous finding,^5^ these rats also showed severe sleep–wake fragmentation due to short duration but not bout number (Figure S2), especially during the light period (Figure S2). Finally, the rats with partial SLDx exhibited a loss of muscle atonia and RBD‐like movements during REM sleep (Figure S3). RBD‐like movements were from simple jerking, twitching to jumping and running characterized by violent and quick movements that were very different from normal movements. Together with unique EEG, RBD‐like movements were very distinguishable from active wake.

These findings indicate that the SLD is necessary and sufficient for REM sleep generation and muscle atonia. In addition, the SLD contributes to stabilization of sleep–wake state including REM sleep state.

### 3.3 Ablation of SLD glutamatergic neurons eliminates REM sleep in mice

**FIGURE 3:**
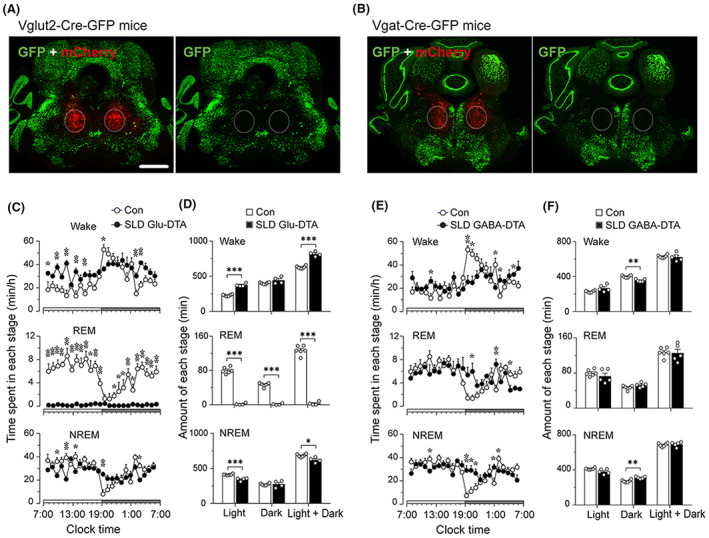
**Ablation of SLD glutamatergic neurons but not GABAergic neurons eliminates REM sleep.** (A) Representative brain sections from control (*N* = 6) and SLD^Vglut2‐DTA^ mice (*N* = 5) show intact and complete loss of glutamate neurons (GFP^+^; green cells) in the SLD. As non‐glutamate neurons do not express Cre and hence are not killed by the Cre‐dependent AAV‐DTA, these neurons express mCherry that marks the injection site (red fluorescence). C and D, respectively, show hourly NREM sleep, REM sleep and wake amounts in light and dark period, and total amounts of NREM sleep, REM sleep and wake in 24 h, light and dark period. The same arrangement of panels for the lesions of SLD VGAT neurons (*N* = 5) is shown by B, E, and F. This figure clearly illustrates that loss of glutamatergic neurons but not GABAergic neurons in the SLD abolishes REM sleep. Scale bar = 500 μm. The time spent in each stage (wake, NREM, and REM sleep) was analyzed using two‐way ANOVA followed by Bonferroni's post hoc test. The amounts of each stage were analyzed using unpaired *t*‐test, *p < 0.05, **p < 0.01, ***p < 0.001.

We next selectively and completely ablated either glutamatergic or GABAergic neurons from the SLD in mice in order to identify the neuronal subset crucial for REM sleep and muscle atonia. For this, we placed bilateral sufficient volume injections of a Cre‐dependent AAV‐DTA (60 nL) into the SLD of Vglut2‐IRES‐Cre‐GFP mice (Vglut2‐DTA^SLD^, *N* = 5) or Vgat‐IRES‐Cre‐GFP mice (Vgat‐DTA^SLD^, *N* = 5). Additional cohorts of these mice received a control vector of AAV‐mCherry (Vglut2‐mCherry^SLD^, *N* = 6 and Vgat‐mCherry^SLD^, *N* = 6) and served as negative controls. All mice were implanted with EEG/EMG electrodes. Four weeks after surgery, we recorded sleep–wake with time‐locked video for 24 h and sacrificed the mice for histological processing. We observed a complete, bilateral, loss of SLD glutamatergic or GABAergic neurons in Vglut2‐DTA^SLD^ and Vgat‐DTA^SLD^ mice, respectively (Figure 3A,B). The lesions avoided a key site for arousal, the PB. The Vglut2‐DTA^SLD^ mice displayed a loss of REM sleep. In addition, these mice showed ca. 30% increase in wake amounts (34.1 ± 1.9 min/h in SLD^Vglut2‐DTA^ vs. 26.2 ± 2.3 min/h in controls, *p* < 0.01) and ca. 20% reduction in NREM sleep (25.8 ± 1.1 min/h in SLD^Vglut2‐DTA^ vs. 28.4 ± 1.9 min/h in controls, *p* < 0.05). The increased wakefulness in SLD^Vglut2‐DTA^ mice resulted from a significant increase in wake bouts (Figure S4A).

The Vgat‐DTA^SLD^ mice did not exhibit amount changes in wake, NREM sleep, or REM sleep (Figure 3). However, these mice displayed an increase in circadian (day–night) difference of wake (55.7 ± 0.7% vs. 49.4 ± 0.9% of control mice) and a decrease in circadian difference of NREM sleep. The Vgat‐DTA^SLD^ mice also displayed sleep–wake fragmentation with increased transitions between NREM sleep and wake, although its fragmentation degrees were much less than those observed in Vglut2‐DTA^SLD^ mice (S4B).

These results indicate that SLD glutamatergic neurons but not GABAergic neurons are necessary for REM sleep generation, but both sets of neurons participate in stabilizing sleep–wake state.

### 3.4 REM sleep elimination increases fear memory

We then investigated the role of REM sleep in consolidation of fear memory using rat model with complete SLDx. We used the following fear conditioning paradigm (see Section 2 for a detailed description). Briefly, during fear acquisition period on day 1, all rats were placed in fear conditioning boxes with white light and 1.0% acetic acid odor (context A) for 180 s. Rats then received three tone/foot‐shock pairing trials (tone: 2000 Hz, 85 dB, 18 s; shock, 0.54 V, 2 s) with 30‐s interval. On day 2, rats were placed in the same box with red light and pine oil odor (context B) for 9 min and 50 s. On day 3, rats were placed in the box with context A with a 120 s pre‐tone period before receiving 5 × 20 s of tone in 30 s intervals, but no foot‐shocks. On day 7, rats were placed again in context A for 120 s pre‐tone and were presented with 180 s tone. These procedures were conducted between ca. ZT 3 and 5.

We first tested whether fear acquisition and recall protocol itself might affect REM sleep. We implanted EEG/EMG electrodes in five rats and 3 weeks later subjected them to day 1, 2, 3, and 7 treatments and recorded EEG/EMG/video during the following night as above. Because daytime stress appears mostly affecting nighttime REM sleep,^26^, ^27^, ^28^ we selected night recording. Compared to the baseline, REM sleep amounts almost doubled during the dark period on all treatment days (day 1, 2, and 7) but returned to baseline levels on day 8 (Figure S5), indicating that fear acquisition, altered contexts, and fear recall all selectively increase REM sleep amounts.

To investigate whether chronic completed loss of REM sleep affects fear expression, we placed large bilateral injections of a volume 120 nL mixture of Cre‐dependent AAV8‐DTA‐mCherry and AAV8‐Cre (*N* = 8 rats) or AAV‐GFP (controls; *N* = 8 rats) into the SLD and performed the above‐described fear conditioning procedures at 3 weeks after the surgery. Importantly, we repeated the day 7 recall procedure once every week for 6 weeks consecutively, and again on week 25 to examine whether the effects are sustained. After completion of the fear conditioning experiments (i.e., 9 months after DTA injections), we implanted the rats with EEG/EMG electrodes and recorded sleep–wake for 24 h (2 weeks after electrode implantation). As expected, all 8 rats injected with AAV‐DTA displayed bilateral loss of SLD neurons and complete loss of REM sleep. These data indicate that SLDx may abolish REM sleep chronically with no signs of REM sleep for at least 9 months.

**FIGURE 4:**
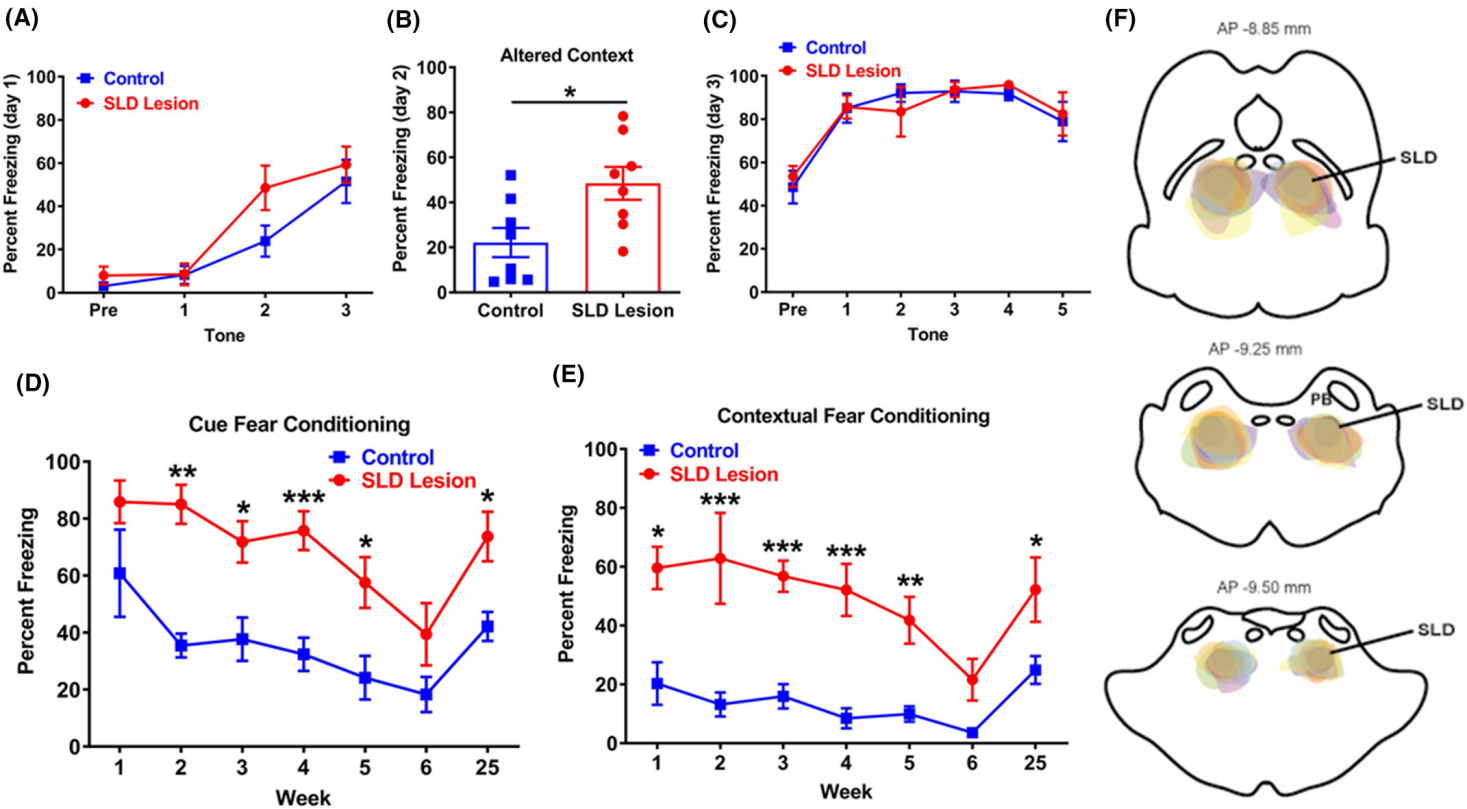
**REM sleep loss enhances fear memory**. To investigate the role of REM sleep in fear memory, we examined and compared the changes between rats with SLD lesions (*N* = 8) and controls (*N* = 8) in fear memory consolidation. Rats with SLD lesions and controls were trained for fear acquisition (three tones paired with three shocks) in context A (day 1), and altered context (context B) (day 2) and five tones alone in context A (day 3) and one long tone alone in context A (day 7 and week 2–6, and week 25). Using freezing time to tones (tone freezing) and pre‐tone (contextual freezing) indirectly determines fear memory consolidation. Compared to control rats, rats with loss of REM sleep show non‐significantly higher freezing responses to the second shock and tone in fear acquisition (day 1) (A) and significantly longer freezing time in altered contextual condition in day 2 (B), but no changes in day 3 (C). From week 1 for contextual fear and week 2 for toned fear to week 5, lesioned rats show significantly higher freezing in contextual and cued condition than control by 2.5 and 1.0 fold, respectively (D and E). In week 6, the differences become not significant. Although weekly contextual and tone exposures progressively reduce contextual and cued freezing response in both control and lesioned group, lesioned group still exhibits much higher contextual and cued freezing than that of control. Between week 7 and 24, no tests were given. In week 25, both groups show again parallel higher level of fear responses than that in week 6, with SLD lesioned rats showing significantly higher freezing responses than the control (D and E). Perfused brains confirm that the lesion borders include the SLD marked by lines but avoids the parabrachial nucleus (PB), shown at three levels in (F). The percent freezing in day 2 was analyzed using unpaired *t*‐test, and others were analyzed using two‐way ANOVA followed by Bonferroni's post hoc test, *p < 0.05, **p < 0.01, ***p < 0.001.

Next, we analyzed the freezing responses in these SLDx rats with chronic loss of REM sleep. On day 1 of the fear conditioning paradigm, the SLDx rats showed higher freezing to the second tone and shock than controls in context A, and the freezing time doubled when they were exposed to the same box with altered context B on day 2 (48.5 ± 7.3% in SLDx rats vs. 22.1 ± 6.5% in controls, *p* = 0.0172), indicating high generalized fearful state. All tests with exception for day 2 were done in the context A. Day 3 test did not result in any significant changes in freezing time. On day 7 (week 1 post‐training), SLDx rats froze significantly more during the contextual period (prior to the tone) but not during the cued period (tone). During week 2–5, SLDx rats consistently showed significant increases in freezing time during both the context (~2.5 fold) and cue periods (~1.0 fold), compared to controls (Figure 4D,E). The freezing times in both groups progressively reduced in parallel and the differences still remained until week 6 (the difference was not significant). Interestingly, when the animals were tested again in week 25, both lesioned rats and controls showed a similar proportional significant elevation in contextual and cued freezing compared to controls (Figure 4D,E).

These data indicate that REM sleep elimination elevates general anxious and fearful state (day 1–2 response) and increases contextual fear memory consolidation (week 1) and retains high fear memory consolidation of context and cue (week 2 and after), with strong preference on contextual fear memory.

### 3.5 Neural circuitry underlying REM sleep‐dependent memory consolidation

Since the hippocampus is highly active during REM sleep^29^, ^30^ and is a principal site for learning and memory processes, we hypothesize that the SLD modulates fear memories through the hippocampus. To test this hypothesis, we first examined how SLD neurons connect the hippocampus by placing unilateral injections of an anterograde tracer, AAV8‐ChR2‐GFP into the SLD (60 nL; *N* = 3 rats), and perfused them after 2 months. In all three rats, the AAV injections (GFP^+^ neurons) were restricted to the SLD, but we found no GFP^+^ axon terminals in the hippocampus or the cortex. We found a large number of GFP^+^ terminals in the medial septum (MS) and the nucleus of the diagonal band (NDB), especially on the cholinergic neurons (identified by ChAT staining) in these regions (Figure S6).

As the cholinergic MS/NDB heavily and almost exclusively projects to the hippocampus and this pathway is responsible for hippocampal activation during wake and REM sleep,^31^ SLD‐MS‐hippocampus circuit may regulate fear memory. We first applied chemogenetic stimulation to the MS and examined sleep–wake changes, EEG fast Fourier transformation (FFT) and fear memory consolidation of contextual and cue fear. Eight rats received non‐Cre‐dependent AAV8‐hM3Dq‐mCherry into the MS (200 nL, UNC core) and implanted with EEG/EMG electrodes. After 3 weeks, rats received saline at 10:00 and CNO at 10:00 in two consecutive days. For fear test, these rats and 8 control rats received fear conditioning in context A and then immediately CNO injection (0.1 mg/kg) around 10:00–11:00 in day 1. All rats were tested in altered contexts (context B) in day 2 and in contextual and cued condition (context A) in day 3. Compared to saline injection, CNO injection did not affect sleep–wake amounts but significantly increased theta power (power ratio, CNO/saline vs. saline/saline) during REM sleep and wakefulness (Figure S7C,D) for about 6 h. Compared to the controls, CNO injection post‐fear training did not alter freezing time on day 2, but significantly reduced freezing time to tones by 15% and contexts by 50% on day 3 (Figure S7E). Given the time period after CNO injection had a very little of wake amounts, it suggests that MS‐induced hippocampal activation of REM sleep post‐fear training reduces contextual and cued fear memory consolidation with strong preference on contextual fear memory.

**FIGURE 5:**
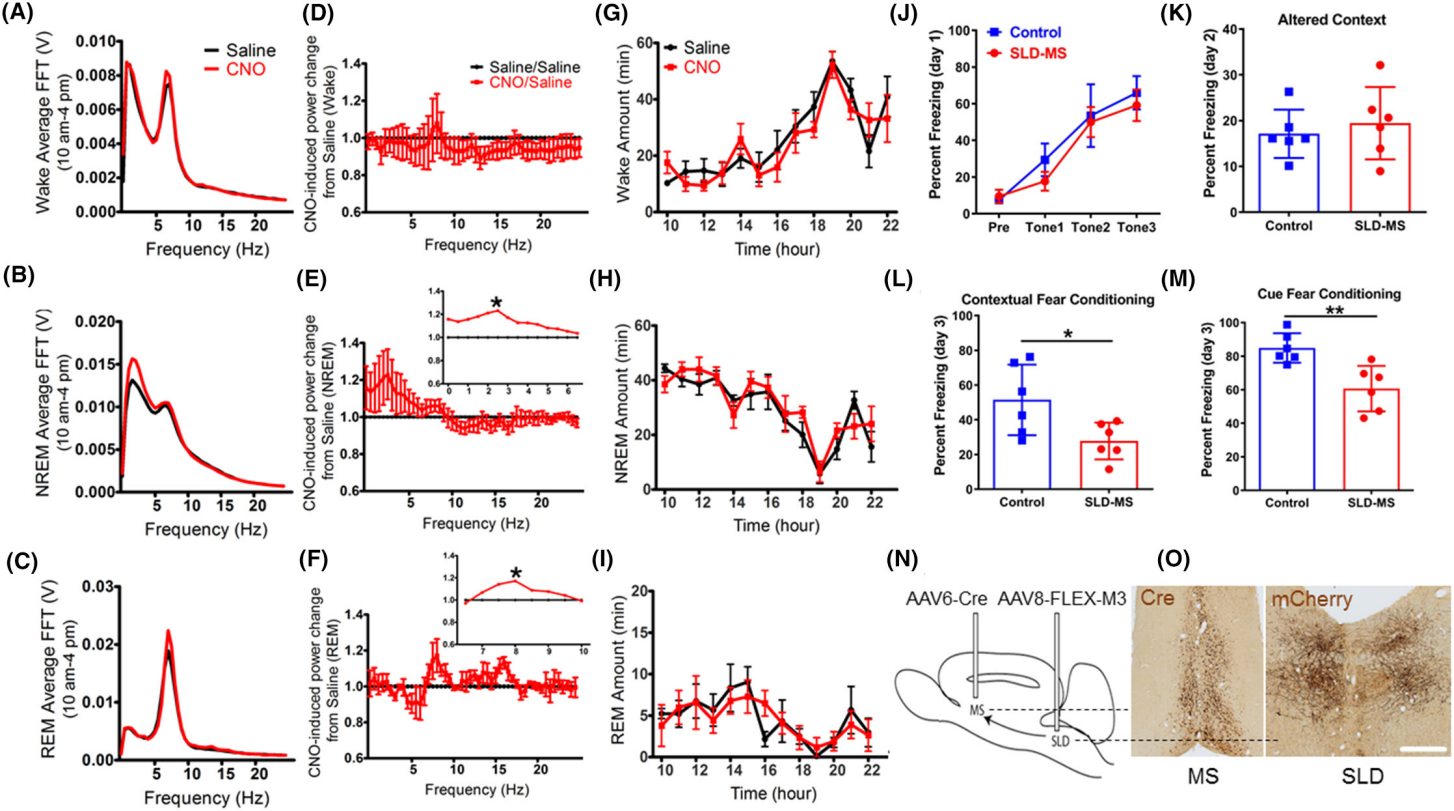
**Chemo‐activation of SLD‐MS pathway reduces fear memory consolidation.** To investigate the role of SLD ascending projection to the MS on fear memory, we injected AAV6‐Cre into the MS and Cre‐dependent AAV8‐M3‐mCherry into the SLD in six rats (N and O). As AAV6‐Cre is retrogradely transported to the SLD, where Cre meets Cre‐dependent AAV8‐M3‐mCherry, resulting in the SLD neurons that project to the MS contains AAV8‐M3‐mCherry. Histology of Cre and mCherry immunostaining confirms Cre in the MS and mCherry in the SLD (N and O). Compared to saline injection, CNO injection at 10:00 am does not alter sleep–wake amounts or its pattern (G–I) but significantly increases delta EEG during NREM sleep and theta activity during REM sleep for about 5 h (A–C, D–F). Compared to control rats, CNO injection does not alter freezing in altered contextual condition (day 2) but significantly reduces contextual fear response by 60% and cued fear memory by 30% in day 3 (J–M). The FFT and amounts of each stage (wake, NREM, and REM sleep) and the percent freezing in day 1 were analyzed using two‐way ANOVA followed by Bonferroni's post hoc test, and the percent freezing in day 2 and day 3 was analyzed using unpaired *t*‐test, *p < 0.05, **p < 0.01. Scale bar = 100 μm.

To be more specific on SLD‐MS projection, we next selectively activated MS‐projecting neurons in the SLD (SLD^MS^) and assessed changes in EEG theta (reflecting hippocampal activity) and fear responses. For selective chemogenetic activation of SLD^MS^ neurons, we bilaterally injected a retrograde AAV encoding Cre (AAV6‐Cre) into the MS and a Cre‐dependent AAV8‐hM3Dq‐mCherry (AAV‐hM3) into the SLD in rats (SLD^MS‐hM3^; *N* = 6) and implanted them with EEG/EMG electrodes. Six rats with sham surgeries (without AAV injection) served as controls (SLD^MS‐sham^). After 3 weeks, we injected saline or CNO (0.1 mg/kg, i.p., to activate hM3‐expressing SLD^MS^) at 10:00 and examined changes in sleep–wake behavior and EEG power spectra. In SLD^MS‐hM3^ rats, CNO did not alter sleep–wake amounts but significantly increased hippocampal theta power during REM sleep and 2.0 Hz delta power during NREM sleep for about 5 h, as compared to saline treatment in the same cohort (Figure 5). For fear memory test, we administered CNO (0.1 mg/kg, i.p.) to SLD^MS‐hM3^ and SLD^MS‐sham^ rats, within 15 min after both groups were trained for fear conditioning (day 1 as described above) around 10:00–11:00. We then assessed freezing behavior on the day 2 and day 3 paradigms. Activation of SLD^MS^ neurons post‐fear training did not affect freezing times in the altered context on day 2 but significantly reduced contextual and cued freezing time on day 3 testing (contextual freezing: 22.45 ± 4.34% vs. 52.49 ± 12.78% in controls, *p* = 0.0338; tone freezing: 59.53 ± 8.49% vs. 86.18 ± 5.47% in controls, *p* = 0.0022) (Figure 5). Day 3 reduction of contextual and cued freezing was about 60% and 30%, respectively. These findings further demonstrate that selective hippocampal activation in REM sleep by the SLD reduces both contextual and cued fear memories with particularly strong preference on contextual fear memory.

## 4 DISCUSSION

The key findings are: (1) SLD glutamatergic neurons generate REM sleep, (2) REM sleep elimination induces fearful (anxious) state and increases fear memory consolidation, (3) fear emotion and memory induce more REM sleep, and (4) REM sleep and SLD via the hippocampus reduce contextual and cued fear memory consolidation with strong action on contextual fear memory.

Our data that the complete loss of REM sleep after SLD lesions and the absence of its recovery even after 9 months prove that the SLD is the sole generator of REM sleep. In absence of the SLD, REM sleep is most likely lost permanently. The residual REM sleep and presence of RBD‐like movements observed in previous lesion studies were likely due to that the some SLD neurons containing low level of glutamate receptors are resistant to ibotenic acid. Conversely, we also found that photo‐stimulation of SLD neurons during NREM sleep facilitated REM transitions, indicating that SLD is sufficient for REM sleep. However, photo‐activation during wake never induced REM sleep transitions, indicating that wake state prevents REM sleep occurrence even under SLD activation.

We previously showed that SLD GABAergic neurons expressed cFos induced by high REM sleep in rats,^5^ confirmed by extracellular recording,^32^ but deletion of SLD GABA neurotransmission did not alter REM sleep or produced RBD‐like movements in mice.^9^ In contrast, loss of SLD glutamate neurotransmission reproduced the REM sleep reduction with sleep–wake fragmentation and RBD‐like movements. We now demonstrate that loss of SLD glutamatergic neurons completely abolished REM sleep, reduced NREM sleep, and increased wakefulness with sleep–wake fragmentation, which is similar to selective REM sleep deprivation in animals.^33^, ^34^ Partial SLD lesions particularly reduced REM sleep duration, indicating that SLD neurons maintain REM sleep. Although loss of SLD GABA neurons did not alter REM sleep amounts, it caused sleep–wake fragmentation that is not seen in deletion of SLD GABA transmission.^9^ The difference may be due to that other neurotransmitters in GABAergic neurons are involved in regulation of REM sleep stability. Likewise, SLD glutamatergic neurons may also contain other neurotransmitters involved in REM sleep generation. Or these GABA and glutamate deletions are incomplete. Sleep fragmentation by lesions of SLD glutamatergic neurons was far more severe than lesions of SLD GABAergic neurons. Thus, SLD glutamatergic neurons are entirely responsible for REM sleep generation; these neurons together with GABAergic neurons consolidate wake–sleep state. As lesions in the SLD or SLD glutamatergic or GABAergic neurons all flattened circadian rhythm of sleep, it suggests that the SLD is involved in circadian regulation of sleep.

Since the discovery of REM sleep, the precise function of this unique sleep state has been under debate. One widely held view is that REM sleep may facilitate implicit learning and memory such as negative emotional memories. However, the findings by sleep deprivation on the REM sleep‐fear memory connection in animals and human are largely inconsistent,^35^ which may be due to stress associated with REM sleep deprivation procedures in those studies as stress of various levels may differently affect fear emotion and memory.^36^ An increase in fear memory consolidation in our animal model of REM sleep elimination strongly supports that REM sleep reduces fear memory consolidation. Similar to chronic REM sleep elimination, acute REM sleep elimination by antidepressants^37^ results in high fear responses in rodents,^38^, ^39^ although these studies did not make connections between REM sleep and fear memory.

It is widely accepted that the hippocampus together with the medial prefrontal cortex and amygdala complex regulates fear learning and memories.^40^ The SLD projects to the MS, the necessary and sufficient driver for the hippocampal activity.^31^, ^41^ Selective lesions of MS cholinergic neurons by 192‐saporin impair fear extinction memories, resulting in high fear expression in rats.^42^, ^43^ We demonstrate that chemo‐activation of the MS and MS afferents from SLD neurons during REM sleep post‐fear conditioning reduced contextual and cued fear memory consolidation. Because of the closeness of the SLD and caudal cholinergic LDT and both projecting to the MS, the stimulated neurons may include LDT cholinergic neurons that project to the MS. However, at this caudal level, there are only sparse cholinergic neurons, and the stimulation mostly comes from the SLD. It is generally believed and demonstrated that the hippocampus controls contextual fear memory but not cued fear memory.^44^ However, there are studies reporting that hippocampal lesions also affect cued fear freezing.^45^, ^46^ Our data showed that REM sleep elimination and hippocampus activation in REM sleep affected contextual fear memory much more than cued fear memory. Nevertheless, cued fear memory was significantly altered. Controls and SLDx rats showed similar high fear memory (ceiling effects) in day 3; however, SLDx rats showed elevated fear responses afterwards, indicating that REM sleep is also critical for fear memory forgetting process. Although SLDx also produced sleep fragmentations, Hunter showed that REM sleep deprivation but not sleep fragmentation impairs fear memory extinction in rats.^47^ Thus, REM sleep elimination may particularly disrupt forgetting process of fear memory. As fear memory and extinction are simultaneously processed in the hippocampus, it is most likely that down‐regulation of hippocampal firing activity during REM sleep^48^, ^49^ regulates both fear memory and extinction. The prefrontal cortex and amygdala that a strong interaction with the hippocampus during REM sleep may also be involved in regulation of fear memory. In short, the hippocampus during REM sleep down‐regulates fear memory consolidation by reducing fear memory formation and promoting fear memory forgetting, with strong preference on contextual fear memory.

Our results appear to contradict a recent study suggesting that theta activity during REM sleep may increase contextual fear memory consolidation.^50^ In that study, selective optogenetic inhibition of MS GABAergic neurons during REM sleep (but not during NREM sleep or wake) post‐fear training reduced REM sleep theta activity in mice, which reduced the consolidation of contextual fear memory but not cued fear memory.^50^ Species (mice vs. rats) and methodological differences (two day test vs. three day paradigm), the specific neurons manipulated (SLD neurons projecting to the MS cholinergic neurons vs. MS GABAergic neurons), and the approach (optogenetics vs. chemogenetics) may have contributed to the differences.

One crucial limitation of fear tests in animals is that it is impossible to separate conscious memory response from subconscious memory responses. Meta‐analysis of human data indicates that REM sleep is particularly critical for suppression of subconscious negative emotions but not emotional recognition memory,^51^ which is also supported by a recent study on REM‐related obstructive sleep apnea.^52^ In accordance, insomniacs display strong physiological responses during fear conditioning and impaired fear extinction.^53^ Negative emotional disorders such as depression, anxiety, attention deficient hyperactive disorder (ADHD), and post‐traumatic stress disorder (PTSD) display short REM sleep latency, high density, and increased amounts of REM sleep.^54^, ^55^, ^56^, ^57^ Our results would interpret that negative emotion and memory trigger REM sleep increase, which in turn mitigates negative emotion and memory.

## AUTHOR CONTRIBUTIONS

Conceptualization: JL, YJW, BG; Methodology: JL, RV, YJW, WJY, CNG, MHQ, DK, JGN, BG; Investigation: YJW, WJY, CNG, MHQ, DK, SQZ, XFY; Visualization: JL, YJW, WJY, CNG, MHQ, DK; Funding acquisition: JL, YJW; Project administration: JL; Supervision: JL; Writing – original draft: JL, MHQ, DK; Writing – review & editing: JL, RV, YJW, JGN, DK, BG.

## FUNDING INFORMATION

This work was supported by the following funding: National Institutes of Health grant R01 NS 061841(JL), National Institutes of Health grant R01 NS 095986 (JL), National Natural Science Foundation of China (32160191).

## CONFLICT OF INTEREST STATEMENT

Authors declare that they have no competing interests.

## Supporting information


Appendix S1

## Data Availability

All data are available in the main text or the Appendix S1.
