# Supplementary material for: Pontine control of rapid eye movement sleep and fear memory
Source: CNS Neurosci Ther. 2023 Feb 16;29(6):1602–14. doi: 10.1111/cns.14123 (PMC10173714; doi:10.1111/cns.14123)
Supplement: Supplementary file 1 — Appendix S1 [file CNS-29-1602-s001.docx]

Supplementary Materials for

**Pontine control of rapid eye movement sleep and fear memory**

Yu Jun Wen, Wen Jia Yang, Chun Ni Guo, Mei Hong Qiu, Daniel Kroeger, Jian Guo Niu, Shu Qin Zhan, Xi Fei Yang, Barbara Gisabella, Ramalingam Vetrivelan, Jun Lu

Correspondence to: lujun@jlu.edu.cn

**This file include:**

Supplementary Figs 1 to 7

Fig. S1

**
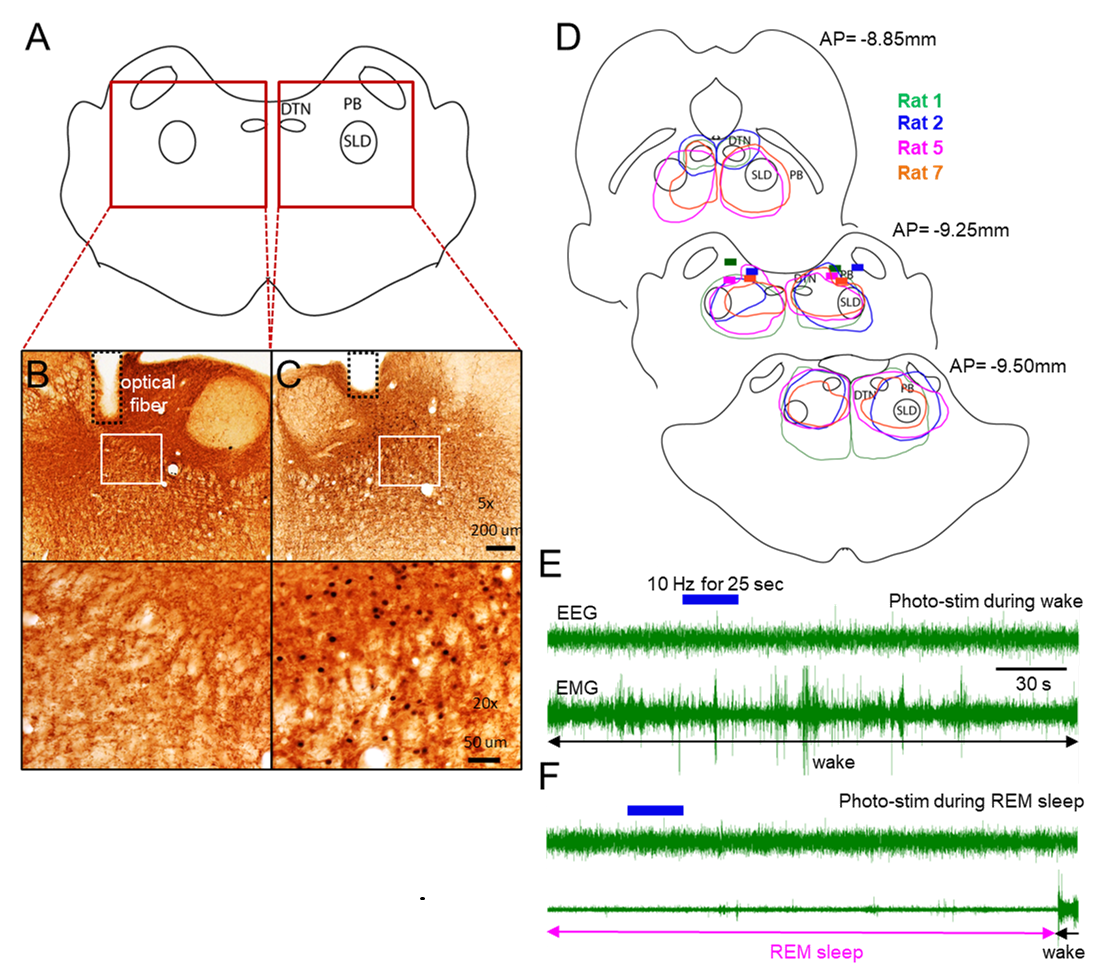
**

**Fig. S1 Photo-activation of SLD induces cFos expressions but fails to alter wake or REM sleep state.** (A-C) Photo-activation for 2 h prior to perfusion induces cFos expression in SLD neurons near the tip of the optical fibers, but sham stimulation has no effect. Brown color marks YFP-expressing neurons and black colored nuclei indicate cFos expression. (D) Mapping of virally transduced neurons and optical fiber placement in each rat. Note: animal ID, fiber placement and viral transfection share the same color. Photo-stimulation during wakefulness (E) or REM sleep (F) does not result in state changes. DTN, dorsal tegmental nucleus; PB, parabrachial nucleus. Scale bars in (C) are 200 µm (overview picture) and 50 µm (enlarged panel).

**Fig. S2**

**
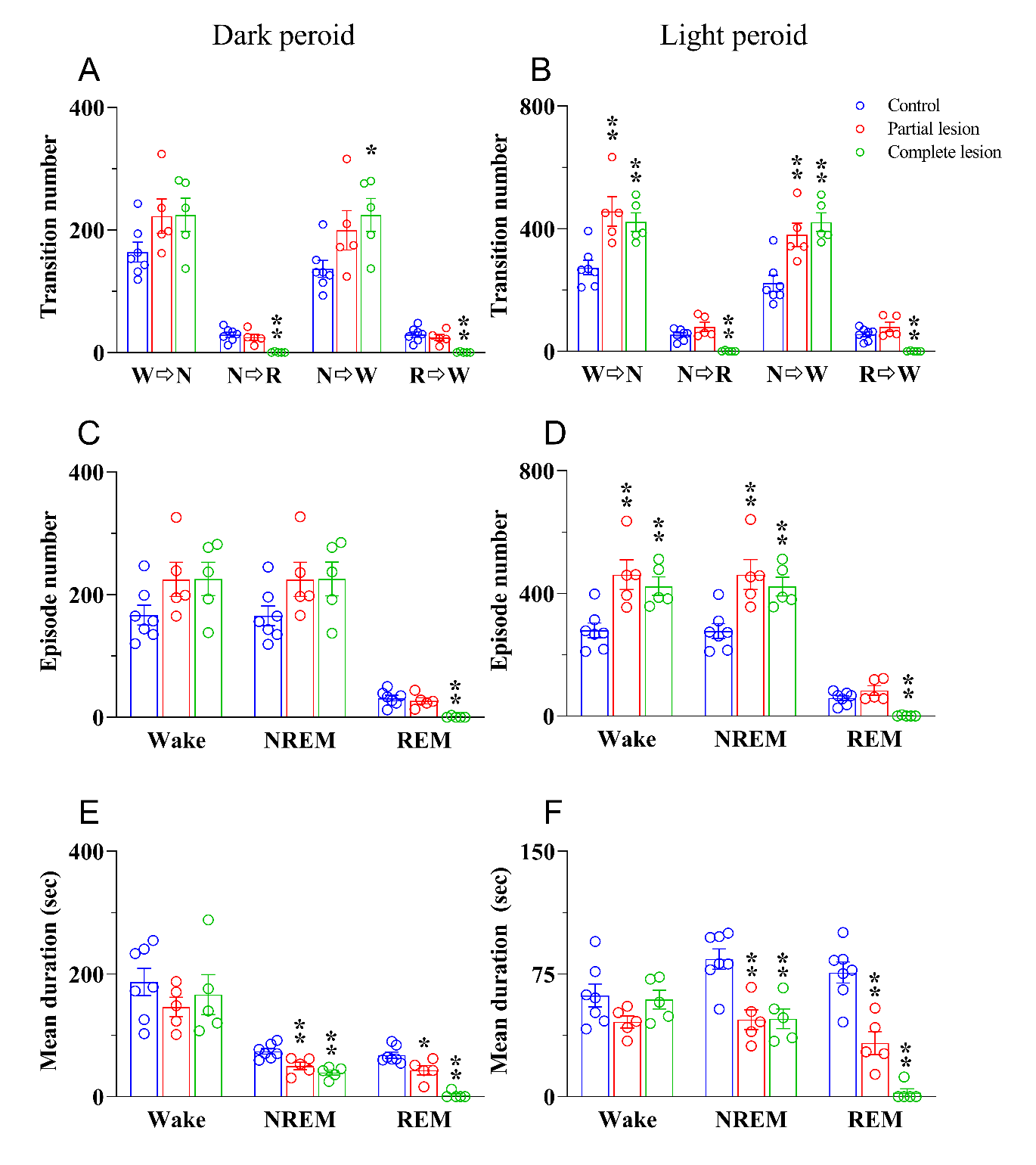
**

**Fig. S2 SLD lesions produce sleep-wake fragmentation.**

Both partial and complete SLD lesions result in frequent transitions between sleep and wake state (A and B) with an increase in sleep and wake bout numbers (C and D) and reduction in sleep and wake durations in dark (19:00-7:00) and light period (7:00-19:00) (E and F). The transition, bout, and duration of wake, NREM and REM sleep were analyzed using one-way ANOVA followed by Bonferroni’s post hoc test, * *P* < 0.05, ** *P* < 0.01.

**Fig. S3**

**
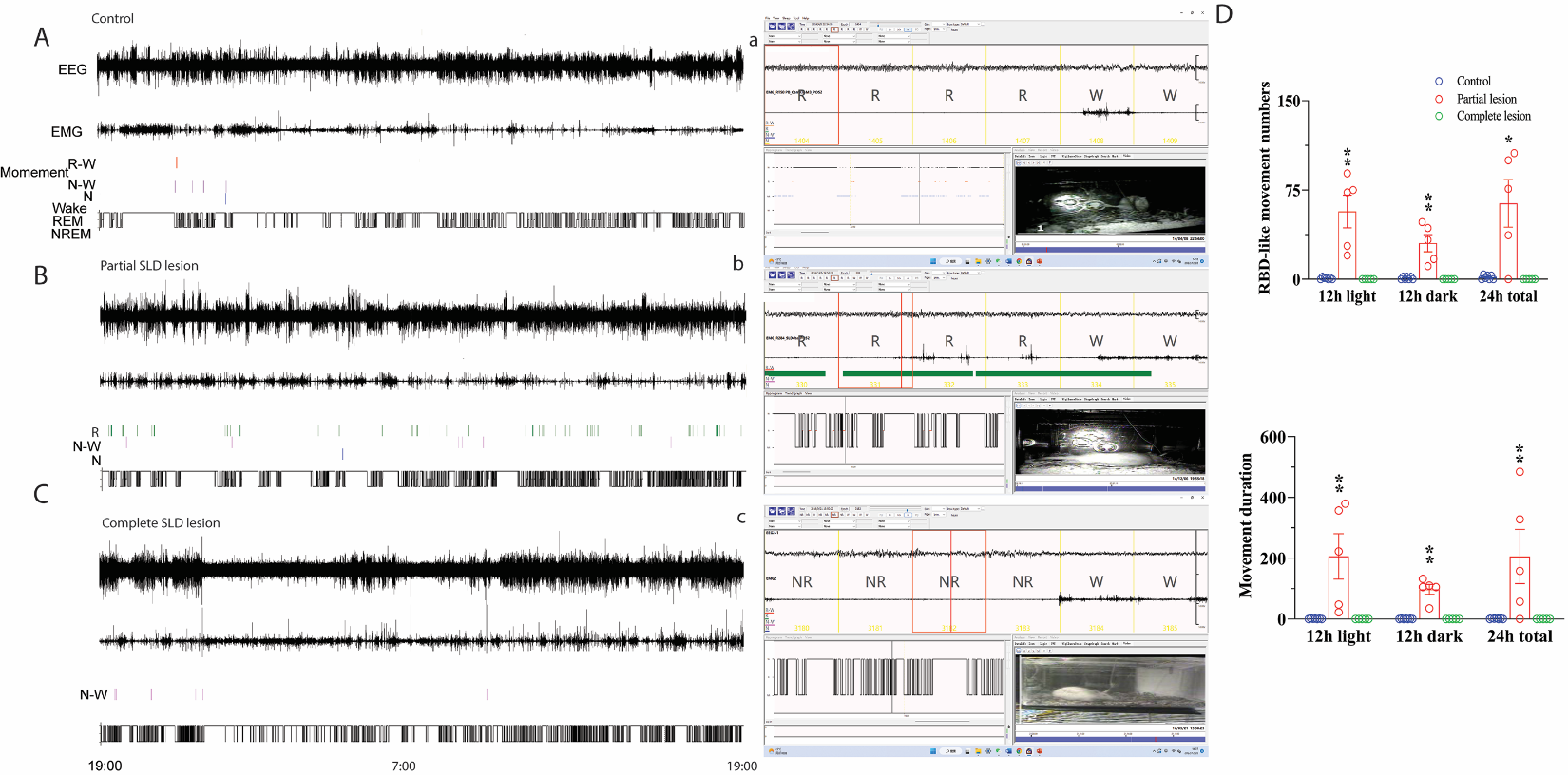
**

**Fig. S3 Hypnograms with RBD-like movements.**

Representative 24 hours hypnograms with sleep associated motor movements of control (A, a), partial (B, b) and complete (C, c) SLD lesions show that a control rat has occasional phasic movements during transitions from REM sleep to wake (R-W) (red vertical line), from NREM sleep to wake (N-W) (purple vertical line) and during NREM sleep (N) (blue vertical line) while bilateral partial SLD lesions produce RBD-like movements (green vertical line) and frequent sleep-wake transitions; complete SLD lesions produce a total loss of REM sleep (and therefore no RBD-like movements) and frequent sleep-wake transitions. Quantified RBD-like movements are shown in (D). RBD-like movements were analyzed using one-way ANOVA followed by Bonferroni’s post hoc test, * *P* < 0.05, ** *P* < 0.01.

**Fig. S4**

**
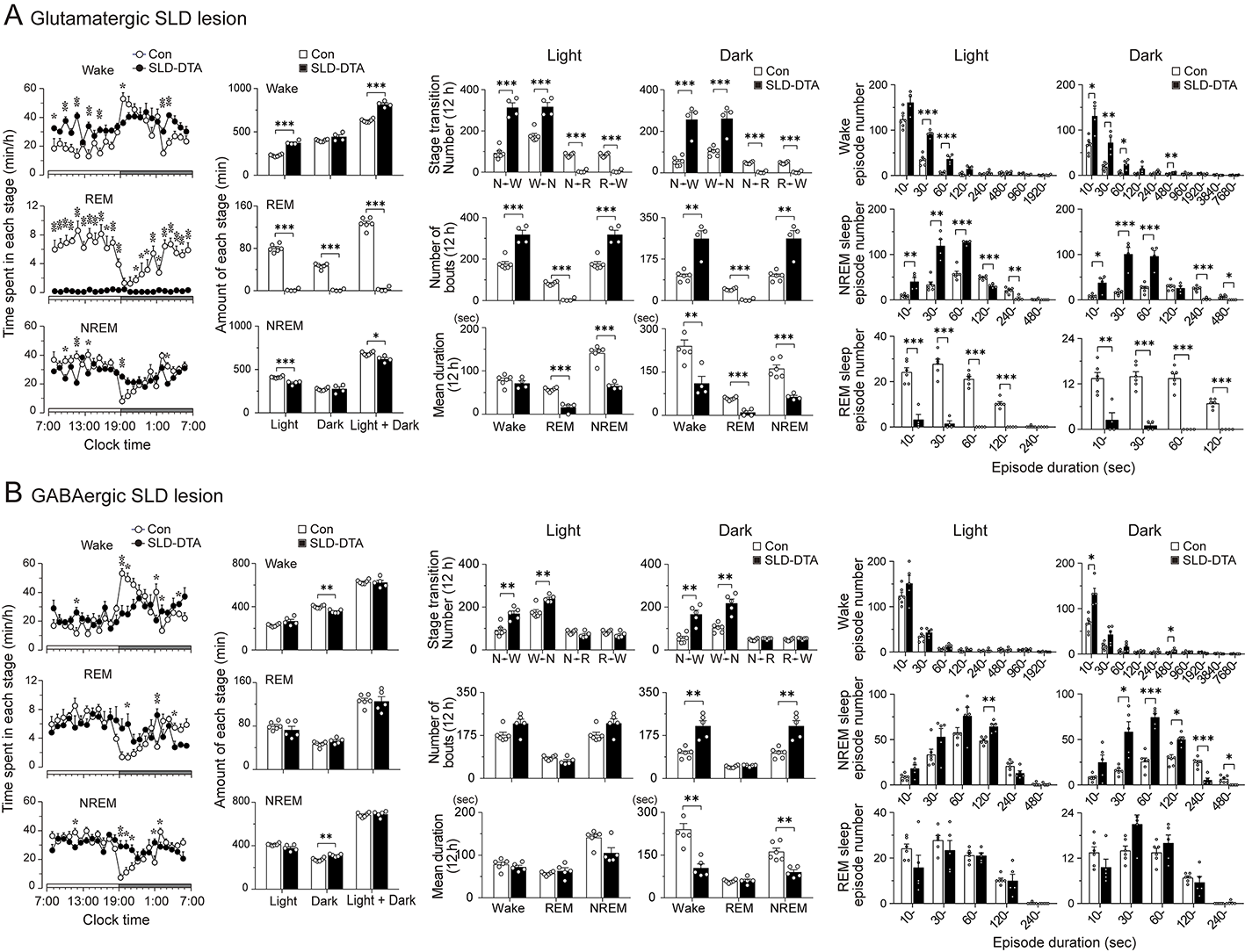
**

**Fig. S4 Effects of selective ablation of SLD glutamatergic and GABAergic neurons on sleep-wake parameters.**

(A) Hourly amounts of wake, REM sleep, NREM sleep, and stage transition analysis in control (N = 6) and SLD^Vglut2-DTA^ mice (N = 5) during the light/dark period and 24 h period. (B) Hourly amounts of wake, REM and NREM sleep and transition analysis in control (N = 6) and SLD^Vgat-DTA^ mice (N = 5) during the light/dark period and 24 h period. Loss of either glutamatergic neurons or GABAergic neurons in the SLD causes sleep-wake fragmentation but only loss of glutamatergic neurons in the SLD eliminates REM sleep. The hourly amounts of each stage were analyzed using two-way ANOVA followed by Bonferroni’s post hoc test. The other parameters of each stage were analyzed using unpaired T test, * *P* < 0.05, ** *P* < 0.01, *** *P* < 0.001.

**Fig. S5**

**
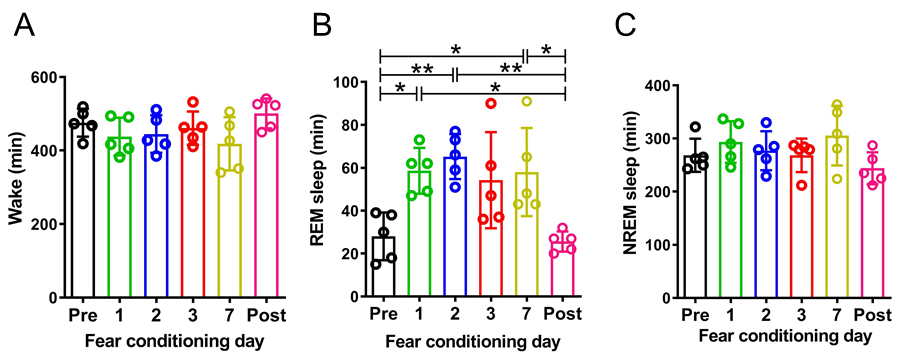
**

**Fig. S5 Fear acquisition and recall selectively increase REM sleep.**

To test how fear learning and memory affect REM sleep, we used one group of 5 rats that went through 1, 2, 3, and 7 day procedure. We recorded EEG/EMG signals for entire night (19:00-7:00) on day 0 (baseline day), 1, 2, 3, 7 and 8 (post-treatment day). All procedures for fear experiments were done around 10:00-11:00. In day 0 and 8, rats were handled during 10:00-11:00. Animals recorded six night EEG/EMG (19:00-7:00) following protocol of the baseline, fear acquisition (day 1), altered contextual exposure (day 2), conditional stimuli (day 3 and day 7) and recovery day. Compared to the baseline, fear acquisition or altered contexts or conditional stimulus selectively and significantly increased REM sleep amounts by two folds. REM sleep returns to the baseline level in the recovery night (B). No significant effects were seen on wake (A) and NREM sleep (C) amounts by fear conditioning and conditioned stimulus. The amounts of wake, REM and NREM sleep were analyzed using one-way ANOVA followed by Bonferroni’s post hoc test, * *P* < 0.05, ** *P* < 0.01.

**Fig. S6**

**
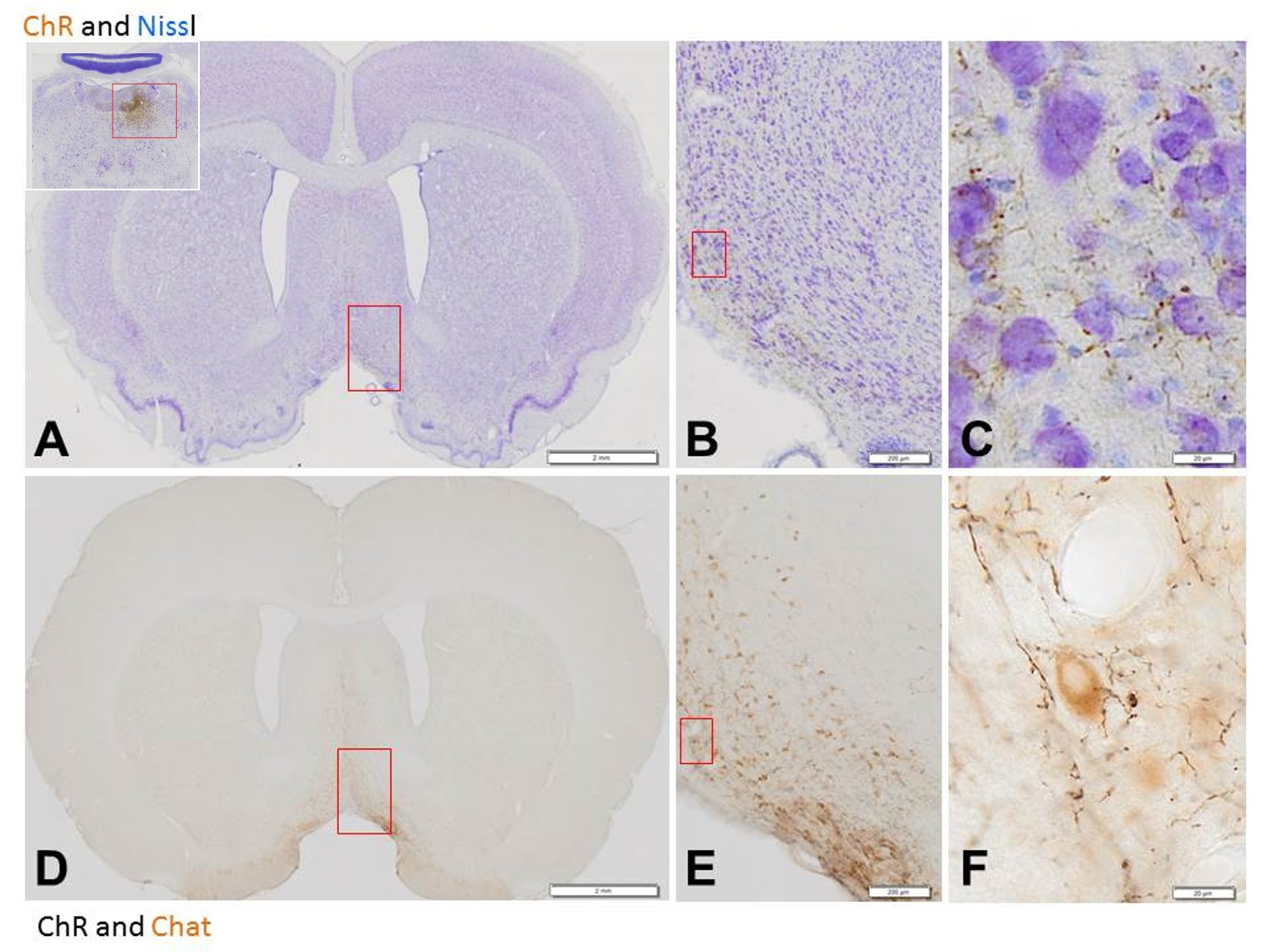
**

**Fig. S6 SLD efferents on the medial septal cholinergic neurons.**

AAV8-ChR2-GFP injection into rat SLD (top left panel in A) shows GFP-labeled terminals (C, F) apposing the cholinergic (ChAT) neurons (F) in the medial septum (MS) and the nucleus of diagonal band (NDB). A-C, immunohistochemical and Nissl staining; D-F, immunohistochemical double staining. Panel B and E are the enlargement of the boxes in panel A and D, respectively. Panel C and F are the enlargement of the boxes in panel B and E, respectively. Scale bars are 2.0 mm (A, D), 200 µm (B, E), 20 µm (C, F).

**Fig. S7**


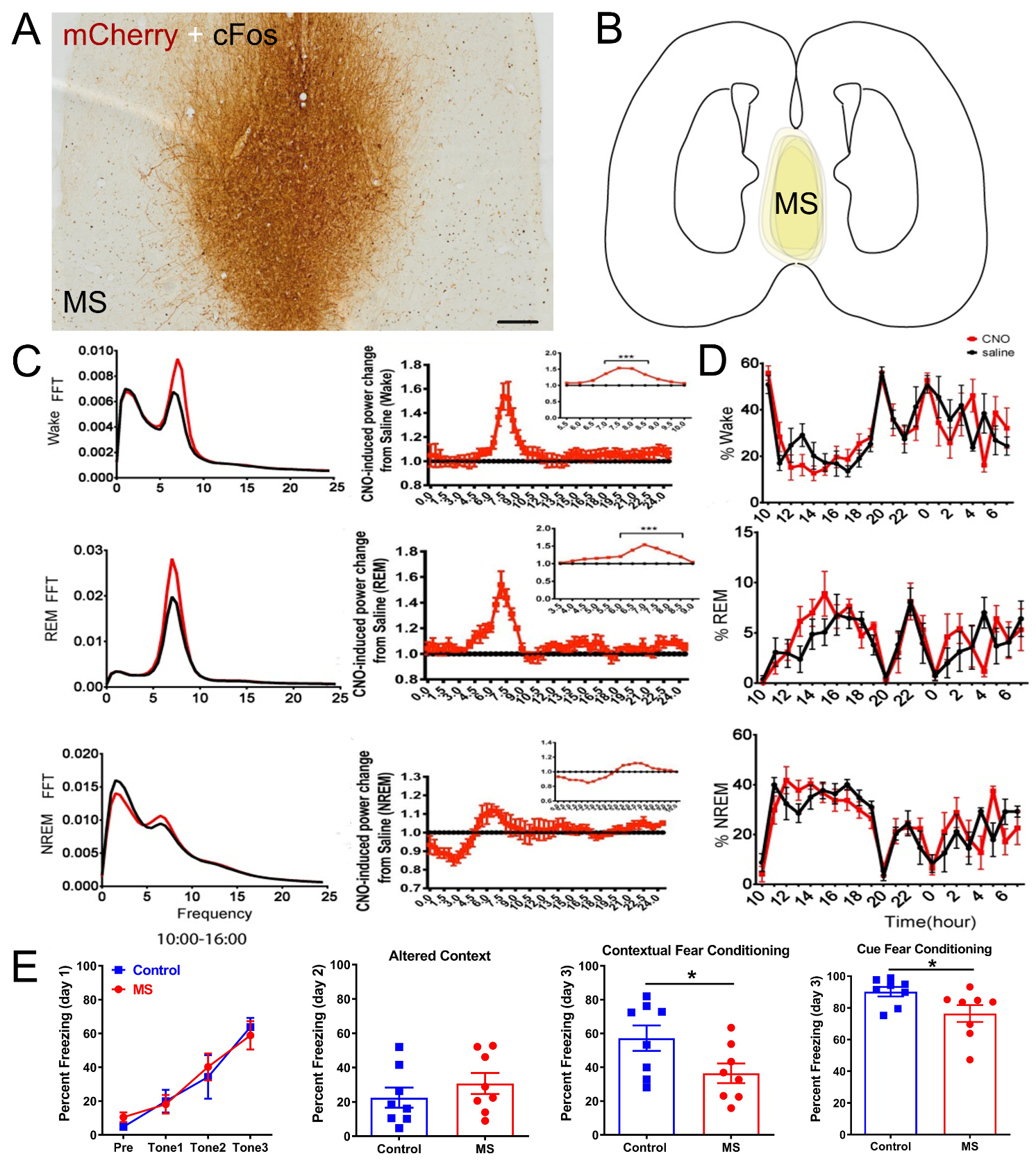


**Fig. S7 Chemo-activation of the MS stimulates the hippocampus during REM sleep and reduces fear memory consolidation.**

To investigate whether chemogenetic stimulation in the MS in sleep increases theta EEG and how it affects fear memory, AAV8-hM3Dq-mCherry (200 nL) injection into the MS (AP: + 0.5 mm, ML: 0 mm, DV: - 6.6 mm) and implanted EEG/EMG electrodes in 8 rats. After three weeks, rats received saline and CNO injection at 10:00 in two consecutive days, during which period EEG/EMG/video were recorded. EEG spectra and sleep-wake amounts were analyzed. These rats and 8 controls (sham surgery) then received conditioning training and immediate CNO injection. In day 2 and 3, contextual and cued freezing were examined. Finally rats received CNO injection and perfused two hours later. The sectioned tissues were labeled with mCherry and cFos. The injection site (MS) was labeled by mCherry and cFos (A) and mapped individually (B). In C, the left panel illustrates average FFT differences of NREM sleep, REM sleep and wake between CNO and saline injection in 10:00-16:00 in the same rats. In the right panel, frequency power ratio changes (CNO/saline vs. saline/saline) were used for statistical analysis. Compared to saline injection, CNO injection significantly increased theta EEG (hippocampal activation) during wake and REM sleep for 6 hours. In D, percentage of NREM sleep, REM sleep and wake per hour after saline and CNO injection at 10:00 are plotted. In E, freezing time is plotted in fear training in day 1, altered context in day 2 and fear memory in day 3. The results combined with FFT data indicate that hippocampal activation during REM sleep after fear training significantly reduced contextual and cued fear freezing time by 50% and 15% respectively, compared to normal REM sleep. Scale bar = 200 µm. The FFT, amounts of each stage (wake, NREM and REM sleep) and the percent freezing in day 1 were analyzed using two-way ANOVA followed by Bonferroni’s post hoc test, the percent freezing in day 2 and day 3 were analyzed using unpaired T test, * *P* < 0.05, *** *P* < 0.001.
